# Lived experiences of parenting-related distress in mothers with gynecological cancer: a descriptive phenomenological study

**DOI:** 10.1186/s12905-026-04745-z

**Published:** 2026-08-03

**Authors:** Dan Liu, Bing Fu, Silan Yang, Quan Shen, Qinjing Yang, Rong Li

**Affiliations:** 1 Department of Neurosurgery, The Third Xiangya Hospital, Central South University, Changsha, Hunan 410013 China; 2 Department of Gynecology, The Third Xiangya Hospital, Central South University, Changsha, Hunan 410013 China; 3 Xiangya School of Nursing, Central South University, Changsha, Hunan 410013 China; 4 Nursing Department, The Third Xiangya Hospital, Central South University, Changsha, Hunan 410013 China

**Keywords:** Gynecological cancer, Maternal practice, Parenting-related distress, Qualitative study

## Abstract

**Background:**

Mothers with gynecological cancer face unique challenges in balancing their maternal role with the demands of diagnosis and treatment. However, their experiences of parenting-related distress during the early phase remain underexplored. This study aimed to explore these experiences.

**Methods:**

A descriptive phenomenological design was adopted. Using purposive maximum variation sampling based on age, cancer type, disease stage, residence, and time since diagnosis, 16 mothers with gynecological cancer were recruited from a tertiary hospital between January and March 2025. Data were collected through face-to-face, in-depth, semi-structured interviews and analyzed using Colaizzi’s seven-step method.

**Results:**

Three main themes emerged: (1) disrupted maternal identity, including cancer shock, interrupted caregiving, and dependency-amplified concern for children’s future; (2) renegotiated maternal practice, through constrained adjustment, family support, and protective silence; and (3) reconstructed maternal meaning, via reframed expectations, child-centered motivation, and restored maternal worth.

**Conclusion:**

Parenting-related distress in mothers with gynecological cancer is a role-specific experience arising from the conflict between illness and maternal responsibilities. Healthcare providers should assess this distress early, support mothers in maintaining their maternal identity, and facilitate family communication and practical caregiving arrangements.

## Introduction

Gynecological cancers represent a substantial and growing public health burden among women worldwide [1, 2]. In China, the incidence and mortality of major gynecological cancers, including cervical and ovarian cancer, have continued to increase, with the annual number of new cases projected to reach 408,300 by 2030 [3]. Meanwhile, the age at onset of some gynecological cancers has shown a downward trend. Taking cervical cancer as an example, its peak incidence age has shifted toward the reproductive years of 15–49, meaning that more women are diagnosed during life stages closely associated with fertility, child-rearing, and family responsibilities [3]. For mothers with dependent children, gynecological cancer therefore constitutes not only a threat to physical health but also a disruption to everyday maternal roles, caregiving practices, and family functioning.

Motherhood is widely understood as both a socially constructed caregiving role and an important component of women’s self-identity [4]. When a mother is diagnosed with cancer, treatment-related physical limitations, psychological distress, and uncertainty about prognosis may undermine her ability to engage in daily childcare and family life, creating tension between the role of “patient” and the role of “mother” [5]. Previous research suggests that having dependent children can add to the psychological and care-related burden of cancer patients. For example, a study by Chien et al. (2022) found that advanced cancer patients with dependent children reported significantly lower health-related quality of life and higher distress levels compared to those without children [6]. Thus, for mothers with cancer, illness-related distress may be intensified by the perceived inability to maintain expected maternal responsibilities.

Within this context, parenting-related distress has been identified as a child-focused form of illness-related suffering [7]. It refers specifically to anxiety and distress arising from impaired maternal functioning in relation to child well-being and family stability [8]. Unlike general cancer-related distress, parenting-related distress centers on mothers’ concerns about their children’s emotional well-being, future development, family stability, and the possible consequences of maternal death or functional impairment. Existing evidence suggests that such distress is an important factor in the psychological adaptation of reproductive-age patients with gynecological cancer and may also influence treatment decisions and adherence by altering patients’ priority assessments [9, 10]. This distress may be especially salient in the early phase after diagnosis and during active treatment, when maternal routines are abruptly interrupted, treatment decisions must be made under uncertainty, and family roles are rapidly reorganized. Broader evidence from pediatric oncology further shows that parenting stress is shaped by psychological, family, and illness-related factors, highlighting the importance of family adaptation and caregiving burden when conceptualizing parenting-related distress [11]. In addition, parenting-related distress may also intersect with broader psychosocial processes involved in cancer adaptation, such as emotional regulation, treatment decision-making, illness-related self-perception, and body-related concerns [12–14]. Therefore, it should be understood not only as emotional suffering, but also as a psychosocial experience involving identity, communication, embodiment, decision-making, and family roles.

Although existing systematic reviews have described the challenges faced by parents with cancer in balancing multiple roles, addressing parenting difficulties, and meeting family needs [15, 16], important gaps remain in relation to mothers with gynecological cancer. First, current evidence on parenting and cancer has focused largely on breast cancer survivors or mixed cancer populations, whereas the experiences of mothers with gynecological cancer remain relatively underexplored, particularly during the early phase of diagnosis and active treatment [17]. This distinction is important because gynecological cancers and their treatments may involve threats to fertility, bodily integrity, femininity, sexuality, and intimate relationships, all of which may shape how women interpret illness in relation to motherhood [18]. Second, previous studies have often examined this issue through broad constructs such as psychological distress, family functioning, or supportive care needs, with less attention to how mothers make sense of parenting-related distress through maternal identity and maternal practice [19]. Moreover, research in the Chinese context remains limited and is still dominated by quantitative approaches, which may not fully capture the layered emotional responses, family communication dilemmas, and meaning-making processes experienced by mothers during the early illness trajectory [20, 21].

Therefore, this study aimed to explore the lived experience of parenting-related distress among mothers with gynecological cancer during the initial diagnostic and treatment period, with particular attention to how maternal identity is disrupted, negotiated, and reconstructed within the family context. Charmaz’s illness and self-identity perspective suggests that serious illness can disrupt individuals’ taken-for-granted sense of self and require them to reinterpret who they are in relation to illness, social roles, and future expectations [22]. This perspective is relevant to the present study because gynecological cancer may challenge not only women’s identity as patients but also their established identity as mothers. It therefore provides a useful lens for understanding parenting-related distress as an identity- and role-based experience, rather than only as a general psychological response to cancer.

## Methods

### Study design

This study adopted a descriptive phenomenological design to explore how mothers with gynecological cancer experienced parenting-related distress during the early phase of diagnosis and treatment. In this study, the early phase was operationally defined as the period within six months after diagnosis and active treatment initiation.

### Theoretical perspective

This study was drew on Charmaz’s theory of illness and self-identity, which emphasizes that serious illness can disrupt an individual’s established sense of self and prompt identity re-evaluation and reconstruction. In the present study, this theoretical perspective was used as a sensitizing conceptual lens during interpretation, rather than as a predetermined coding framework. The coding process and theme development remained grounded in participants’ accounts, while Charmaz’s perspective helped interpret how parenting-related distress was embedded in maternal identity disruption and the ongoing negotiation between being a patient and being a mother.

### Setting and participants

Participants were recruited and interviewed at Xiangxi Autonomous Prefecture People’s Hospital, a tertiary Grade-A hospital in Hunan Province, China, between January and March 2025, using purposive sampling with a maximum variation strategy. Variation was sought in age, educational level, marital status, occupational type, disease stage, and time since diagnosis in order to capture a broad range of maternal experiences. This study was conducted during the Hunan Provincial High-level Hospital Paired-Assistance Programme for medical institutions in resource-limited regions. Under this programme, The Third Xiangya Hospital, Central South University served as the supporting hospital, and Xiangxi Autonomous Prefecture People’s Hospital served as the recipient hospital. The main research team members were affiliated with The Third Xiangya Hospital and participated in this study during the paired-assistance period.

Eligible participants were women with a pathologically confirmed diagnosis of gynecological cancer, such as cervical, ovarian, or endometrial cancer, who had primary parenting responsibility for at least one minor child aged 18 years or younger, were cognitively intact, able to communicate verbally, and willing to participate with written informed consent. Women with severe psychiatric illness or cognitive impairment that would interfere with the interview were excluded, as were those who were currently pregnant or breastfeeding. Potentially eligible participants were first identified by gynecological oncology clinicians according to the inclusion and exclusion criteria. The research team then reviewed eligibility, approached eligible women in person, explained the study purpose, interview procedures, voluntary nature of participation, and confidentiality measures, and invited them to participate. Sample size was determined by informational sufficiency and data saturation. Recruitment ceased when no substantively new meanings or thematic insights emerged in two consecutive interviews, and the research team agreed that the thematic structure was adequately developed. A total of 18 potentially eligible participants were introduced to the research team by the clinicians. Among them, two women refused to take part: one reported that she could not spare time because of continuous childcare obligations, and the other stated that she was not comfortable with the topic of the interview. No further refusals occurred, and the final sample consisted of 16 participants.

### Data collection

An initial semi-structured interview guide was developed based on a comprehensive review of domestic and international literature and integration of clinical experience. The guide explored the impact of illness on family life, changes in childcare arrangements, current parenting challenges, perspectives on the maternal role, and desired support. To ensure appropriateness and feasibility, the guide underwent pilot testing with two gynecological cancer mothers. Following feedback, the guide was revised and refined. The finalized interview guide primarily included the following questions: (1) Since your diagnosis, what changes has your illness brought to your family life and your childcare responsibilities? (2) How do you feel your role as a mother has changed since becoming ill? (3) Currently, what are the greatest difficulties or worries you face regarding caring for your child(ren)? (4) How do you typically cope with or manage these worries? (5) What kind of support or help do you most hope to receive from healthcare providers, family, or society? (6) Is there anything else you would like to add or share regarding this interview?

The interviews were conducted by DL and BF, both registered nurses with clinical nursing backgrounds who had received prior training in qualitative interviewing and had been involved in cancer-related research. They had no pre-existing therapeutic or caregiving relationship with the participants, which helped reduce potential social desirability bias during interviews. Before each interview, the interviewers introduced their research roles, explained the purpose of the study, and reassured participants that they could pause or withdraw from the interview at any time without affecting their treatment. After written informed consent was obtained, interviews were arranged at a time when participants were physically and emotionally able to take part. Individual face-to-face semi-structured interviews were conducted in a private and quiet setting within the hospital. Interviews lasted approximately 30–60 min, were audio-recorded with permission, and were transcribed verbatim. Field notes were recorded during and immediately after interviews to document non-verbal cues, emotional responses, contextual details, and preliminary analytic reflections. During the interviews, probing and follow-up questions were used to elicit richer descriptions and clarify meanings. Data collection and preliminary analysis were conducted concurrently so that emerging themes could inform subsequent interviews and allow the researchers to judge data saturation.

### Data analysis

Data analysis was conducted by R L and QJ Y. All interview recordings were transcribed within 24 h following each session. To explore parenting-related distress among mothers with gynecological cancer, the interview data were systematically analyzed using Colaizzi’s seven-step phenomenological approach. The analytical procedure involved the following: repeatedly reviewing the full set of transcripts to achieve familiarity with the data; extracting significant statements pertinent to the phenomenon under study; formulating meanings and coding these statements; organizing codes into thematic clusters; integrating the resulting themes into an exhaustive description of the phenomenon; and distilling the fundamental structure of the experience. Finally, this essential summary was returned to participants to seek their validation and feedback [23]. To enhance analytic transparency, two researchers independently coded the transcripts and iteratively compared their interpretations. Any discrepancies were resolved through discussion until consensus was reached, and theme definitions were continuously refined through repeated reference to the original transcripts. Throughout data collection and analysis, reflexive awareness was maintained to critically examine how the researchers’ clinical and academic backgrounds might shape interpretation, thereby enhancing confirmability.

### Trustworthiness

Following Lincoln and Guba’s criteria, trustworthiness was strengthened throughout the research process. Credibility was supported by purposive maximum variation sampling, probing during interviews, member checking, and repeated research team discussions. The synthesized findings were shared with five participants for member checking. They confirmed that the themes accurately reflected their experiences, and no major modifications were made to the thematic structure. Dependability and confirmability were enhanced through independent coding, comparison of interpretations, reflexive awareness, and repeated checking of themes against the original transcripts. To support bracketing, the researchers kept reflexive memos to document their preconceptions related to oncology care, motherhood, family caregiving, and illness-related distress. Emerging interpretations were repeatedly checked against the original transcripts and discussed within the research team to reduce the influence of researchers’ clinical and academic assumptions.Transferability was supported by detailed descriptions of the study setting, participant characteristics, sampling process, and the use of representative quotations.

### Ethical considerations

This study was conducted in accordance with relevant guidelines and regulations, including the ethical principles of the Declaration of Helsinki. It was carried out during a clinical support collaboration between The Third Xiangya Hospital, Central South University and Xiangxi Autonomous Prefecture People’s Hospital. Participants were recruited and interviewed at Xiangxi Autonomous Prefecture People’s Hospital, where the data were collected. Therefore, the study was reviewed and approved by the Medical Ethics Committee of Xiangxi Autonomous Prefecture People’s Hospital (Ethics Approval Number: EC-LCKY2024047). All participants volunteered to take part and provided written informed consent. All recordings and transcripts were anonymized, and identifying information was removed during data processing and reporting.

## Results

### Demographic characteristics

Sixteen eligible patients with gynecological cancers participated in in-depth interviews. Their baseline characteristics were summarized in Table 1. Participants had a mean age of 45.3 years (range: 34–54). Half had completed primary education, and most resided in rural or township areas. Occupations varied, with farming being the most common. Cervical cancer was the predominant diagnosis (62.5%), followed by ovarian cancer (31.3%). Most cases were diagnosed at stage I (56.3%). Participants were diagnosed between August 2024 and January 2025 and were interviewed between January and March 2025. All participants were interviewed within six months after diagnosis, during diagnostic work-up or active treatment initiation, consistent with the operational definition of the early phase used in this study.

**Table 1:** Participants’ characteristics

Participant ID	Age (years)	Education Level	Marital Status	Residence	Occupation	Cancer Type	Stage	Date of Diagnosis
P1	48	Junior high school	Married	Town	Farmer	Cervical cancer	IB1	2024.10
P2	34	Primary	Married	Rural	Farmer	Cervical cancer	IIB3	2024.10
P3	37	College	Married	Urban	Tour guide	Cervical cancer	IB2	2024.09
P4	50	Primary	Widowed	Rural	Farmer	Cervical cancer	IIB	2024.09
P5	43	Primary	Married	Rural	Factory worker	Cervical cancer	IIB	2024.12
P6	48	Primary	Married	Rural	Farmer	Cervical cancer	IIB	2024.11
P7	54	Junior high school	Divorced	Urban	Unemployed	Cervical cancer	I	2024.10
P8	47	Primary	Married	Rural	Farmer	Ovarian cancer	I	2024.12
P9	48	Primary	Married	Rural	Farmer	Cervical cancer	IIB	2024.08
P10	46	Primary	Widowed	Town	Unemployed	Cervical cancer	III	2024.10
P11	46	College	Married	Urban	Civil servant	Ovarian cancer	III	2024.12
P12	44	Junior high school	Divorced	Town	Self-employed	Ovarian cancer	IIIC	2024.09
P13	41	Junior high school	Married	Town	Unemployed	Ovarian cancer	IA	2025.01
P14	42	Bachelor’s degree	Married	Urban	Laboratory technician	Endometrial cancer	IA	2025.01
P15	46	Primary	Married	Rural	Farmer	Ovarian cancer	I	2025.01
P16	51	Senior high school	Married	Town	Unemployed	Cervical cancer	IIA1	2024.10

### Semi-structured interview results

The analysis generated three interrelated themes: disrupted maternal identity in parenting-related distress, renegotiated maternal practice within the family system, and reconstructed maternal meaning through children’s future. Participants shared a common concern for their children, but their experiences differed in emotional intensity, family support, children’s dependency needs, and perceived ability to maintain maternal responsibilities. These differences indicate that parenting-related distress was a dynamic and context-dependent experience rather than a single emotional response.

### Theme 1: disrupted maternal identity in parenting-related distress

Participants described the diagnosis and early treatment period as a profound disruption to their sense of themselves as mothers. Illness affected not only their emotional security but also their ability to carry out everyday maternal responsibilities, resulting in fear, guilt, and heightened concern for their children’s future. This theme captured the initial rupture between participants’ established maternal identity and their newly imposed identity as patients.This theme comprised three sub-themes.

### Cancer shock and child-centered mortality anxiety

Following diagnosis, many participants described intense fear, shock, and a child-focused sense of mortality. Their distress was often expressed not only as fear of cancer itself, but also as fear of leaving young children behind. For these mothers, the meaning of mortality was closely tied to the imagined consequences of their absence from their children’s lives.

P7: " It felt unbearable, as if my whole world had collapsed. I didn't even feel I could go on with my life."

P13: “I felt extremely upset and scared. Hearing it was cancer was terrifying—especially because my child is still so young. I can’t bear the thought of leaving them.”

P14: “I was afraid… very afraid.” Her eyes filled with tears; after a long silence, she began to cry and could no longer continue speaking.

P15: “(Tears welling up) I was afraid. I had cancer before and underwent surgery, and now a cyst was found. I was at work when I noticed bleeding. When I went to the bathroom, there was so much blood—it was terrifying. I went straight to the hospital for an ultrasound, and the doctor told me I had a very large cyst.”

P16: “I can’t control my emotions when talking about illness and death, because I truly do not want to die.”

For some participants, this initial intensity appeared to give way to emotional numbing, exhaustion, or a muted expression of distress over time. This apparent calm did not necessarily indicate the absence of distress; rather, it often reflected resignation, fatigue, or an attempt to contain emotions in front of family members.

P1: “(My emotions) haven’t changed much. I’m sick, and there’s nothing I can do about it (followed by prolonged silence and quiet tears).”

P8: “(Fear, worry, frustration) — none of these. I’ve come to accept it. The only thing on my mind is not to become a burden to them.”

P9: " Getting old is just getting old. I don’t dwell on it anymore. If death comes, I can only accept it. If I am meant to live longer, I will live a few more years; if my time is short, there is nothing I can do but accept it.”

P16: “I thought it was an early stage. When I saw the bleeding, I knew it wasn’t good, but I assumed it was early, so I wasn’t too frightened. Later at the hospital, tests showed it was stage II–III, and that’s when I started feeling anxious — the anxiety was unavoidable at the time. Now, with time, it’s gotten a little easier. Over time, you just grow weary.”

### Interrupted caregiving and maternal incapacity

A central source of distress was the disruption of everyday maternal practice. Hospitalization, treatment, and physical weakness limited participants’ ability to provide daily care, emotional companionship, and household support, making illness visible not only as a medical condition but also as a disruption to the caregiving practices through which they understood themselves as mothers.

P1: “Yes, it definitely affects me as a mother… in many ways.” When asked whether this involved companionship, financial support, and emotional communication, she nodded in tears and said, “All of these.”

P11: “Once I was hospitalized, I could no longer care for my child. The way his father handled things at home is… beyond words… When it comes to meals, his father just throws something together or buys whatever is convenient—it’s definitely not as good as when I cook.”

P8: “I worry about the huge costs of treatment and also that I can’t take care of my child anymore.”

As mothers shifted from caregivers to care recipients, many described feelings of incapacity and guilt. This was especially painful when children had to take on caregiving responsibilities, as the reversal of care roles challenged participants’ sense of maternal competence.

P7: “Look, now I can’t even take care of myself. How can I fulfill my responsibilities as a mother? I’ve even become an extra burden for my child. Emotionally, I’ve put pressure on him too—my son took care of me in the hospital for two months during that time.”

P10: “(My son) was originally in Shanghai. After I got sick, he came back to take care of me because there’s no one else at home. His father passed away more than ten years ago.”

### Dependency-amplified concern for children’s future

For participants caring for children with substantial dependency needs, parenting-related distress was intensified by worry about the child’s future survival and daily functioning. Unlike more general hopes for children’s development, their worries centered on who would care for the child if the mother’s illness worsened, making future-oriented distress more concrete and immediate.

P6: “What worries me most is him (my youngest son who is mute). He can’t do anything on his own — he can’t cook, can’t wash clothes. I’ve always done everything for him. So since I got sick, I’ve been thinking that once I’m back, I need to apply for subsistence allowance for him, so he can at least have a few hundred yuan each month to get by.”

P16: “My biggest concern is for my child (with Down syndrome). He can’t do anything by himself. What will happen to him in the future?”

### Theme 2: Renegotiated maternal practice within the family system

Beyond the initial disruption caused by diagnosis and treatment, participants described ongoing efforts to renegotiate motherhood within the family system. These experiences involved adapting maternal responsibilities under constrained conditions, relying on family support, and navigating difficult patterns of illness-related communication. This theme reflected participants’ attempts to preserve maternal involvement despite reduced physical capacity, while also managing the emotional and practical consequences of illness within the family.

### Constrained adjustment of maternal responsibilities

Although illness limited their physical and emotional capacity, participants did not simply relinquish their maternal role. Instead, they described efforts to remain involved as mothers while also trying to reduce the burden placed on other family members. This negotiation was reflected in lowered expectations, partial role adjustment, and, at times, consideration of withdrawal from daily family life. These accounts showed a tension between wanting to remain needed and fearing that continued dependence on the mother would increase family burden.

P13: “My expectations for them are not high. I only hope they grow up safe and healthy. Beyond that, I don’t have many thoughts—just that they can take care of themselves and become self-sufficient. Growing up healthy is enough; I don’t ask for much, just that they can support themselves.”

P16: “I am considering moving back alone to live in the countryside, as I still have a house there. Currently living with my youngest son, they rely on me for everything—absolutely everything depends on me.”

### Family support for maternal role continuity

Family members were an important source of support during illness. Participants described both practical assistance, such as financial help and help with daily living, and emotional support through companionship, reassurance, and sustained presence. Such support helped some mothers maintain a sense of continuity in their maternal role by reducing treatment-related pressure and stabilizing family life. However, family support varied in availability and quality, and these differences influenced how mothers were able to adjust and maintain their maternal role during illness.

P4: “My brother has always taken great care of me. He has a successful career and is very capable. If I need money, he provides it; if I lack anything, he gives it. So, no matter what happens, I don’t worry. I also have a sister who is employed and well-off, and she shows me exceptional care.”

P5: “My family cared about me and were very attentive. When I was in the county hospital, my younger sister stayed with me the whole time. With their support, I felt much better and stopped dwelling on it.”

P8: “I have five siblings, and they all show deep concern for me. When my sister learned about my illness, she immediately transferred ten thousand yuan. Others sent two thousand, five thousand, and even my nieces and nephews contributed as well.”

P11: “Especially my sister—since my own parents are no longer here, I have three sisters and one brother. After I returned, she came to my home every single day, preparing breakfast, lunch, and dinner for me, and visited daily.”

### Protective silence and emotional distance

Participants described family communication about illness as cautious, limited, and emotionally restrained. A recurring pattern was protective silence, in which both mothers and family members avoided explicit discussion of illness in an attempt to protect one another from additional distress. However, this pattern could also reduce emotional exchange and, in some cases, contribute to withdrawal or conflict. Protective silence therefore operated ambivalently: it was intended to preserve family stability and prevent emotional harm, but it could also leave mothers and children without opportunities for open emotional communication.

P3: “I haven’t told them. My older son is 17 and in his second year of high school, so I didn’t tell him. The younger one is still too young to understand; telling him wouldn’t make a difference.”

P14: "(Shaking her head) I have not told my son. He only knows that I am in the hospital. I think he may have sensed something, but I still have not told him."

As observed in P4: " Just between you and me, they must be worried about me, but they won’t show it in front of me. There’s no change in their emotions, but they want me to fully recover before leaving the hospital.” This reflects a culturally embedded pattern of emotional restraint where family members avoid expressing worry directly to protect the patient.

P2: “(After learning about my illness) I felt depressed and didn’t want to talk. I was scared inside and avoided speaking with others. I hardly communicated with them (my children)—even less after getting sick.”

P13: “It came out during an argument. I hadn’t planned to tell them at first. The two children, one 17 and the other 14, are both in their rebellious phase. During a heated quarrel, when they wouldn’t listen, I told them, ‘It’s because of you that I didn’t go see the doctor earlier. The diagnosis is cancer, and it might not be curable.’ The older one was completely stunned.”

### Theme 3: Reconstructed maternal meaning through children’s future

Despite uncertainty and suffering, participants also described efforts to reorient meaning through motherhood. Rather than abandoning the maternal role, they redefined what mattered for their children, drew motivation from their children’s future, and relied on others’ affirmation to rebuild a sense of personal worth. This theme reflected a movement from disruption toward meaning reconstruction, in which motherhood remained both a source of distress and a source of psychological endurance.

### Reframed expectations for children’s well-being

Participants described a shift in what they hoped for their children. Expectations that may once have emphasized future achievement became more focused on immediate well-being, physical health, and the child’s ability to live independently. This reframing suggested that illness narrowed mothers’ expectations from long-term achievement to more fundamental hopes for safety, health, and self-sufficiency.

P2: “I just hope they grow up well and learn to be independent. Whatever they want to do, I will support them. I do not want to stop them or disappoint them.”

P9: “If you ask me now what worries or hopes I still have, I don’t really have any such thoughts or wishes. But to say I have none isn’t entirely true — I do hope they are all well. I hope my husband and children stay healthy.”

P12:”What worries me most is that my illness might recur. If this happens, things will become difficult. As for my son, I mainly hope that he will be healthy and grow up well. As for what happens next, I can only let him go his own way.”

P16: “I hope to see my children grow up gradually. I hope they can become independent, manage their lives well without relying on me, and I hope they will get regular health check-ups in the future and stay healthy.”

### Child-centered motivation for treatment endurance

For many participants, their children became a central source of motivation for enduring treatment and continuing life. In this sense, motherhood functioned as an anchor of meaning under illness, with survival imagined in relation to remaining present for the child’s future. Unlike the reframing of expectations, this sub-theme emphasized children as an immediate reason for treatment persistence and psychological endurance.

P7: “During my hospitalization, doctors on rounds would tell me, ‘You’re very strong; keep up the treatment for your child’s sake.’ I often feel like a burden, but hearing that from the doctors made me feel that my perseverance still has meaning, as if I regained some strength.”

P8: “Now my children tell me to focus on treatment. They say that even if we have no money, they will borrow money for my treatment. Since they support me like this, I have to take the treatment seriously.”

### Restored maternal worth through external recognition

Illness often weakened participants’ sense of worth, particularly when they felt they were no longer fulfilling their maternal responsibilities as before. Encouragement and recognition from family members, friends, and healthcare professionals helped them feel seen, supported, and reassured that their efforts still mattered. Such recognition partly repaired the loss of maternal competence by affirming that mothers’ previous and ongoing efforts remained valuable despite illness-related limitations.

P9:“When I was in the hospital for treatment, the doctors and nurses often encouraged me and reminded me that I was still a good mother. That made me feel that I still mattered”.

P10: “Sometimes I feel I haven’t fulfilled my responsibilities as a mother, but my family and friends encourage me, saying I’ve already done my best. Their comfort makes me feel a bit better.”

## Discussion

This qualitative study explored parenting-related distress among mothers with gynecological cancer during the early phase of diagnosis and treatment. The findings suggest that parenting-related distress is not merely a general psychological response to cancer, but a role-based and temporally situated experience shaped by disruptions to motherhood. Specifically, participants experienced disrupted maternal identity, renegotiated maternal practice within the family system, and reconstructed maternal meaning through their children’s future. These findings extend previous understandings of cancer-related distress by foregrounding maternal practice—that is, how illness interrupts everyday caregiving, family responsibilities, and mothers’ sense of competence and self-worth. By focusing on the initial diagnostic and treatment period, this study highlights a critical transitional period in which mothers must simultaneously cope with the shock of diagnosis, make treatment decisions, reorganize childcare arrangements, and manage uncertainty about their children’s future. Therefore, parenting-related distress among mothers with gynecological cancer can be understood as a role-specific experience arising from the conflict between illness-related limitations and maternal responsibilities.

### Disrupted maternal identity in parenting-related distress

Our findings show that death anxiety and guilt were closely tied to the interruption of everyday maternal caregiving. Hospitalization, treatment, and physical weakness limited mothers’ ability to provide daily care, maintain family routines, and accompany their children emotionally. In this sense, cancer was experienced not only as a medical diagnosis, but also as a disruption to the ordinary practices through which participants understood themselves as mothers. Although previous studies have documented parenting concerns and psychological distress among cancer patients with minor children [10, 24], our study extends this evidence by showing that, for mothers with gynecological cancer, parenting-related distress was experienced as a rupture in maternal identity.

From the perspective of Charmaz’s illness and self-identity theory, serious illness can disrupt a person’s established sense of self and create a loss of continuity between the previous self and the ill self. In this study, the loss of caregiving capacity challenged participants’ sense of maternal competence and self-worth. Because maternal identity is an important component of women’s self-concept [25], illness-related impairment of the maternal role may create a deeper sense of role disruption. Thus, the distress described by participants reflects a rupture between “being a mother” and “being a patient”: mothers were physically placed in the role of care recipients while emotionally continuing to define themselves through caregiving responsibilities. In the Chinese family context, where motherhood is closely connected with caregiving responsibility and family obligation, the inability to perform expected maternal duties may further destabilize family functioning and intensify mothers’ feelings of guilt, inadequacy, and diminished self-worth [26].

### Renegotiated maternal practice within the family system

The present study further shows that family support was not merely a background resource, but a relational context in which maternal practice was renegotiated during illness. Although family support helped some participants reduce treatment-related pressure and preserve maternal role continuity, its impact varied according to the availability and quality of support and the way illness-related emotions were communicated within the family. Thus, family support should be understood not only as practical assistance, but also as part of family relationships and communication patterns that shape how mothers adjust caregiving responsibilities and maintain maternal identity during illness.

A particularly important finding was the pattern of “protective silence.” Participants and their family members often avoided direct discussion of illness, prognosis, or emotional distress in an effort to protect one another from further psychological burden. This finding is especially meaningful in the Chinese and broader East Asian cultural context, where family harmony, emotional restraint, and avoidance of burdening others are often valued in illness communication. This protective silence can also be understood through the lens of traditional gender roles, where women in Chinese families are often expected to prioritize family harmony and emotional containment over individual expression. In the present sample, protective silence should also be interpreted in relation to participants’ rural or township backgrounds and relatively low educational levels. These contextual factors may shape access to cancer-related information, confidence in discussing prognosis, and preferred ways of expressing concern within the family. Previous studies on cancer communication in Asia and end-of-life communication in Chinese families have similarly shown that patients and families may conceal information or suppress emotions to preserve hope and reduce distress [27, 28]. Our study adds that protective silence also occurs in the context of motherhood and parenting-related distress: mothers may withhold illness-related information from children to protect them, while children and other family members may also conceal worry to protect the mother.

Although protective silence was rooted in care, it had ambivalent consequences. On the one hand, it helped maintain temporary family stability and prevented children from being exposed to overwhelming information. On the other hand, it could limit emotional exchange, increase mothers’ sense of isolation, and delay family conversations about practical caregiving arrangements. Therefore, protective silence represents a culturally embedded family coping pattern that can both support and hinder emotional connection. This finding has important implications for oncology nursing practice. Healthcare professionals should provide culturally sensitive communication guidance that helps mothers and families balance protection, honesty, emotional expression, and practical planning.

### Reconstructed maternal meaning through children’s future

Our findings suggest that children’s future became a central pathway through which mothers reconstructed meaning after diagnosis. From the perspective of Charmaz’s illness and self-identity theory, serious illness disrupts the continuity of the self and requires individuals to renegotiate who they are in relation to illness [22]. In this study, participants’ maternal identity was not simply weakened by cancer; rather, it was reworked through their continuing concern for their children. Even when mothers could no longer fully perform previous caregiving roles, imagining their children’s health, independence, and future life allowed them to preserve a sense of purpose and maternal continuity.

This process also reflects meaning-making after traumatic illness. While Duran and Cetin (2024) examined meaning reconstruction in the context of grief, our findings suggest that for mothers with gynecological cancer, meaning reconstruction is not solely retrospective, but is also future-oriented and deeply intertwined with children’s developmental milestones and life events [29]. Meaning reconstruction was not expressed as an abstract search for the reason behind cancer, but was grounded in concrete maternal concerns: whether children could live well, be cared for, and become independent. In this sense, children’s future functioned ambivalently as both a source of anxiety and a source of endurance. This helps explain why parenting-related distress and treatment motivation coexisted among participants. Supportive care should therefore not only relieve distress, but also help mothers identify feasible ways to maintain maternal involvement, affirm the continuing value of their maternal role, and strengthen family and professional support during the initial treatment period.

### Clinical implications

These findings suggest that oncology nurses should assess parenting-related distress early after diagnosis, especially among mothers with minor children, limited family support, single-parent families, or children with high dependency needs. Assessment should extend beyond general psychological distress to include maternal role disruption, childcare concerns, family communication difficulties, practical caregiving needs, and worries about children’s future. Family-centered support should help mothers maintain feasible involvement in childcare, redistribute caregiving responsibilities, and communicate with children in a gradual, developmentally appropriate, and culturally sensitive manner. For families characterized by protective silence, nurses should facilitate balanced communication rather than simply encouraging full disclosure.

Practical and emotional support should be integrated. Mothers experiencing financial strain, limited childcare resources, or concerns about dependent children may benefit from referral to social workers, community resources, financial assistance, and family counseling services. New strategies, including decision-support tools, emotional regulation interventions, mindfulness-based or body-focused approaches, digital follow-up platforms, online psychoeducation, and remote psychosocial support, may help identify and respond to parenting-related distress [12–14]. However, these approaches should complement rather than replace face-to-face assessment and should be implemented with attention to patients’ digital literacy, privacy concerns, cultural preferences, and access to technology.

## Limitations

Several limitations should be acknowledged. First, all participants were recruited from a single province in central China, which may limit transferability to mothers from other cultural contexts. Although maximum variation sampling was used, participants were relatively homogeneous in regional and sociocultural background. Therefore, the findings should be interpreted with consideration of the Chinese cultural context, in which motherhood, caregiving responsibility, and family obligation may have particular meanings. Variations in family structures, healthcare systems, and cultural norms regarding motherhood and illness disclosure across different regions of China (e.g., urban vs. rural, coastal vs. inland) should be explored in future studies.

Second, this study focused primarily on patients’ perspectives and did not systematically include the views of spouses, children, or other family members. As parenting-related distress is embedded in family relationships and communication patterns, the absence of family members’ perspectives may limit a fuller understanding of how such distress is shared, concealed, or negotiated within the family system. Future research could include participants from multiple centers and regions, adopt longitudinal qualitative or mixed-methods designs, and incorporate the perspectives of spouses, children, and other family members to explore how parenting-related distress evolves, is communicated, and is negotiated within the family system.

## Conclusion

This study reveals that parenting-related distress in mothers with gynecological cancer during early diagnosis and treatment is a multidimensional, role-specific experience. It manifests as disrupted maternal identity, renegotiated maternal practice, and reconstructed maternal meaning. These findings underscore the need for a family-centered, holistic approach to care that goes beyond symptom management to address patients’ maternal roles, childcare concerns, and family communication needs. By providing early assessment, psychological support, and practical resources, healthcare providers can help these mothers navigate the dual demands of illness and motherhood, ultimately fostering better psychological adjustment and quality of life.

## Data Availability

The data that support the findings of this study are available on request from the corresponding author. Data are not made public due to privacy and ethical restrictions.
